# Cation Transporters of *Candida albicans*—New Targets to Fight Candidiasis?

**DOI:** 10.3390/biom11040584

**Published:** 2021-04-16

**Authors:** Marina Volkova, Anastasia Atamas, Alexey Tsarenko, Andrey Rogachev, Albert Guskov

**Affiliations:** 1 Moscow Institute of Physics and Technology, Institutskiy Pereulok, 9, 141700 Dolgoprudny, Moscow Oblast, Russia; volkova.mb@phystech.edu (M.V.); atamas.aa@phystech.edu (A.A.); aleksey.spitsin@phystech.edu (A.T.); 2 Joint Institute for Nuclear Research, 141980 Dubna, Moscow Oblast, Russia; 3 Groningen Biomolecular & Biosciences Institute, University of Groningen, 9747 AG Groningen, The Netherlands

**Keywords:** pathogen, candida, cation transporters, drug target, structure, structural biology

## Abstract

Candidiasis is the wide-spread fungal infection caused by numerous strains of yeast, with the prevalence of *Candida albicans*. The current treatment of candidiasis is becoming rather ineffective and costly owing to the emergence of resistant strains; hence, the exploration of new possible drug targets is necessary. The most promising route is the development of novel antibiotics targeting this pathogen. In this review, we summarize such candidates found in *C. albicans* and those involved in the transport of (metal) cations, as the latter are essential for numerous processes within the cell; hence, disruption of their fluxes can be fatal for *C. albicans*.

## 1. Introduction

*Candida albicans* is the prevalent pathogenic microorganism among the yeast fungi, colonising humans and causing opportunistic infections, generally termed as candidiasis. In humans, *C. albicans* is a part of normal vaginal and gastro-intestinal flora, and over 80% of the human population is colonized with it [1]. Normally, these fungi and the host live in commensalism; however, under certain circumstances, such as immune deficiency, prolonged antibiotics treatment, chemotherapy, malnutrition, and others, *C. albicans* may switch from commensal to the pathogenic state (see Mayer et al. [2] for the review on the pathogenicity mechanisms in *C. albicans*). The oral candidiasis (also termed thrush) is the most common form of candidiasis [3] diagnosed in humans and is typically treated by application of topical anti-fungal drugs such as nystatin or fluconazole in severe cases [4,5]. The second most common is the vulvovaginal candidiasis (or a vaginal thrush) [6], caused by excessive growth of *C. albicans* in the vagina, which is also the second most common vaginal infection (after bacterial infections). It is estimated that at least 70% of women experience vulvovaginal candidiasis during their life [7], with about 5–8% developing the recurrent form of a disease. The typical treatment is based on topical application of cream or suppositories of clotrimazole or nystatin [4].

In immunocompetent patients, the majority of cases are treated well with topical or oral medications; however, in immunocompromised patients, there is a high risk of a systemic infection. Currently, *C. albicans* is the most common hospital-acquired fungal and eukaryotic pathogen in the world. It is ranked the third most causative agent of sepsis in the United States, with about 24 cases per 100,000 patients and a mortality rate of over 40% [8,9]. It can also form biofilms on surfaces of implants and transplanted organs, often in alliance with *Staphylococcus aureus*, hence increasing the mortality. It is estimated that over 45 million medical devices are implanted each year in the United States alone and more than 50% of nosocomial infections are related to these implants. In cases of systemic infection, the typical treatment includes either oral or intravenous administration of fluconazole, echinocandin, or amphotericin B [4].

Unfortunately, there is a steady increase in the number of registered cases of candidiasis annually, caused by the development of resistance to anti-fungal drugs, wider application of immunosuppression therapies, and the global increase in hospital treatments. Within just the USA, the annual treatment costs are estimated to be over a billion USD [10]. Furthermore, the treatment of fungal infections typically has a large number of side effects, as there is a substantial overlap between targets in fungi and homologous targets in humans.

Therefore, it is imperative to define the set of promising targets for further investigations of the structural basis of such adaption and resistance in *C. albicans* in order to develop novel, more selective antifungal medications targeting this important pathogen.

Potent antibiotics selectively inhibit pathogens without significant harm to the host organisms. Around 50% of currently used antibiotics target a ribosome, with the other half targeting either membrane proteins (typically receptors or transporters/channels) or soluble proteins involved in biosynthesis pathways. The main mechanism of action of the current antifungal drugs [11] is either inhibition of the ergosterol synthesis, which is the main component of fungal cell wall (azole drugs), or increasing the membrane permeability via pore formation, leading to ion leakage and cell death (polyenes drugs, such as nystatin and amphotericin B); the latter drugs come with a relatively long list of possible side effects [12,13]. Recently, a novel class of anti-fungal antibiotics has been developed, namely echinocandins [14], which target the β-1,3-D-glucan synthase enzyme, thus interfering with the cell wall synthesis. Unfortunately, the resistance to these drugs is also on the rise and, furthermore, they have poor oral bioavailability and might be embryotoxic.

Therefore, the development of novel anti-fungal medications with higher efficiency and less side effects is highly desirable and rational drug design (when the structural information on a target is known) is one of the most efficient ways to do this.

As with every cellular organism, *C. albicans* needs to transport multiple solutes across its semipermeable membranes; however, as most solutes are charged and bulky, dedicated membrane transporters are necessary. Therefore, interfering with the transport systems might be disruptive for the cell either as a result of insufficiency (inhibition of import) or as a result of overabundance (inhibition of export). Metals, both alkali and ‘heavier’, play an important role in the life cycle of *C. albicans*; moreover, in many cases, these metals are linked to pathogenicity and survival. As metals cannot be synthesized, interfering with its transport can be a promising route to control the growth of microorganisms.

Below, we summarize the most promising targets among (metal) cation membrane transporters for future drug development against *C. albicans*.

## 2. Alkali and Alkali Earth Metals

### 2.1. Ammonium Transporting Systems

Despite ammonium (NH_4_^+^) not being a metal, it behaves very much like an alkali metal between the size range of potassium and cesium. Ammonium is a preferred nitrogen source for many microorganisms and fungus *C. albicans* is no exception. In favorable environments, ammonium is taken up by low affinity non-specific transporters (typically in the form of NH_4_^+^, which is deprotonated to NH_3_ [15] or freely diffuses across the membrane [16]). However, under nitrogen starvation conditions, previously repressed pathways for utilization of alternative nitrogen sources are activated. Consequently, both enzymes (such as secreted aspartic protease Sap2, which digests the proteins in the extracellular environment) and specialized transporters are expressed [17,18]. In *C. albicans,* the specific ammonium transporters are MEP (methylammonium permease) proteins (see Table 1 for unique identifiers of all proteins discussed in this work), which belong to the 

Amt/Mep/Rh family of transporters, present in all kingdoms of life [19].

Interestingly, in addition to the transport function, the ability to sense ammonium levels is ascribed to Mep2 protein, hence termed *transceptor*, as it not only transports the substrate, but also senses it to activate downstream signaling pathways [20,21,22]. The acquirement of such a function is probably dictated by the fact that *C. albicans* cannot easily sample the availability of ammonium in the environment, but instead, intracellular ammonium presence controls morphogenesis and gene expression of proteins responsible for nitrogen utilization [23]. This additional function of Mep2 can be an explanation for why its expression is much higher than that of Mep1, albeit the former one is apparently more efficient in transport [24]. This is not unique for *C. albicans* and was also demonstrated in *S. cerevisiae* [25].

Under nitrogen starvation (and many other environmental challenges, such as the presence of serum, elevated CO_2_ concentrations in host tissues [26], or capture by macrophages), *C. albicans* undergoes a morphogenetic switch from normal budding yeast to filamentous pseudohyphal growth. This morphogenetic switch is associated with the virulence of fungi [27,28,29,30,31]. There is a large body of evidence suggesting that the overexpression of Mep2 promotes such morphogenesis. Whereas both Mep proteins (Mep1 or Mep2) are sufficient to enable growth in low ammonium concentrations [24], only Mep2, as shown in both *C. albicans* and *S. cerevisiae*, triggers the filamentation [24,32].

**Figure 1:**
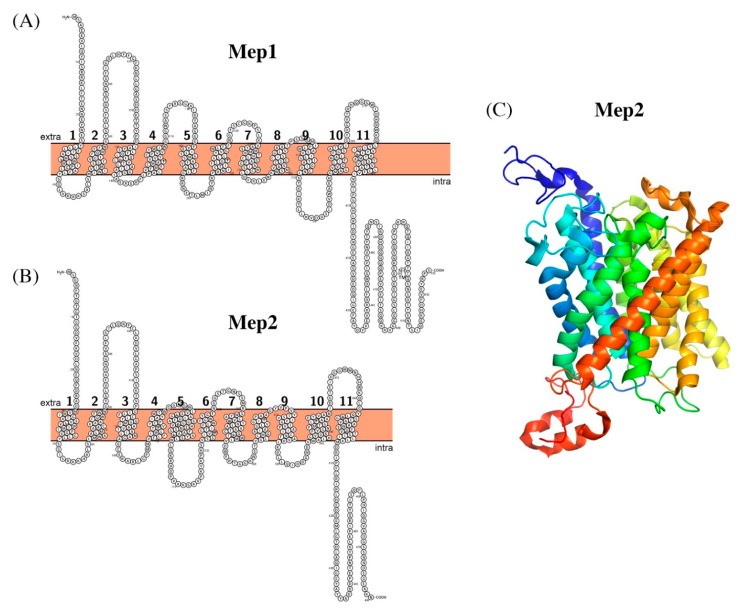
Schematic representation of the secondary structure of Mep1 (**A**) and Mep2 (**B**). Crystal structure of *Candida albicans* Mep2 (**C**); PDB ID: 5AEZ, colored in rainbow, from blue (N-terminus) to red (C-terminus).

Mep1 and Mep2 are 535 and 480 amino acids long, respectively, and both contain 11 transmembrane segments, with a short extracytoplasmic N terminus, and an extended C-terminal cytoplasmic domain (Figure 1). Proteins assemble in functional homotrimers [22]. Mep1 and Mep2 share ~36.5% identical residues.

Several mutagenesis studies have indicated that transport and filamentation functions are strongly coupled in Mep2 [20,29,30,31,32], with an important contribution of the C-terminal domain, as partial truncations of it can cause either hyper-filamentation or completely abolish it [20,32].

Interestingly, as seen in Xenopus oocytes, the transport via Mep1 is electrogenic, whereas it is electroneutral via Mep2, indicating that only Mep1 is capable of co-transporting H^+^ [32].

Based on the high-resolution structures (~1.5 Å resolution) of Mep2 from *C. albicans*, van den Berg et al. proposed a transport mechanism for these transporters [18]: in the dephosphorylated state, intra-monomeric interactions between C-terminal domain and intracellular loop 3 are not present and the translocation pathway is blocked. A single phosphorylation event causes allosteric conformational changes in the C-terminal region of all monomers, which brings it in close contact with the intracellular loop, triggering the opening of the pathway.

Ammonium transceptors such as Mep2 have been proposed as excellent antifungal targets [33] thanks to their essential role in filamentation, their accessibility as cell surface proteins, and a low sequence identity (~17%) with a human homolog; however, no drugs targeting these proteins are currently available.

### 2.2. Sodium Transporting Systems

Alkali metals are omnipresent and play an important role in the maintenance of membrane potential in all kingdoms of life. This is achieved by optimal concentrations of alkali metals in the cytoplasm of cells, and there are numerous transport systems involved in the uptake or removal of cations [33,34]. In yeast, one of such proteins is Cnh1, which is involved in the exchange of intracellular sodium for extracellular protons [35]; hence, it is also involved in the maintenance of intracellular pH. Furthermore, in yeast, including *C. albicans* alkali cations contributes towards the growth and virulence by regulating adhesion and cell-surface hydrophobicity, as well as cell morphology [36,37]. Hence, such systems can be potential drug targets.

**Figure 2:**
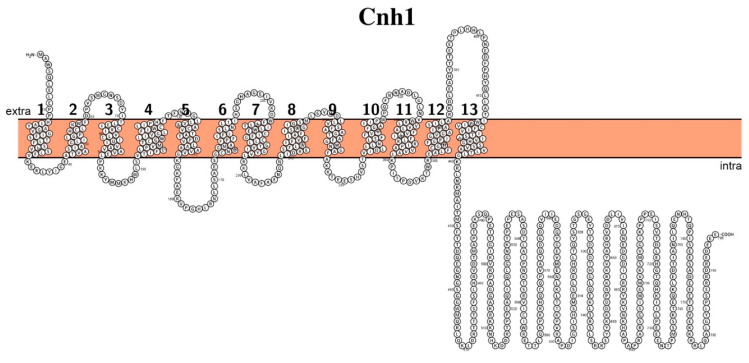
Schematic representation of the proposed secondary structure and topology of Cnh1.

Cnh1 is expressed in the plasma membrane of *C. albicans*, is comprised of 796 amino acid residues (Mw ~89 kDa), and has 13 predicted transmembrane segments (Figure 2). Despite the preposition that the major substrate for Cnh1 is sodium ions, it has rather a broad substrate specificity and additionally is capable of transporting K^+^, Rb^+^, and Li^+^ [38].

Indeed, high intracellular concentrations of sodium ions are toxic for *C. albicans*; therefore, Na^+^ is constantly expelled outside the cell [39] and Cnh1 was proposed to play a major role in this process [40]. Surprisingly though, the knockout strains with *C*nh*1* deletion maintained their normal growth. Further experiments revealed that Cnh1 plays an important role rather in K^+^ and Rb^+^ tolerance, with a marginal contribution to Na^+^ tolerance [34].

Currently, the structure of *C. albicans* Cnh1 is not available, although the membrane embedded part is probably structurally well conserved within the yeast according to the multiple sequence alignment analysis; e.g., it shares 98.6% sequence identity with Nha1p antiporter from *S. cerevisiae*. However the C-terminal part (located in cytoplasm) is not conserved and is highly variable in size from 400 to 800 amino acid residues [38,41]. The exact role of such a variation is not entirely clear, but this domain is predicted to have a regulatory function as this part of a protein reveals numerous putative phosphorylation sites [42]. Furthermore, there are indications that, in some species, C-terminal domain might be disordered, which nevertheless can undergo disorder-to-order transition upon binding with regulatory proteins. This in turn will affect the interactions between this domain and the rest of the protein (embedded in the membrane), hence modulating its activity [41].

In the absence of any Na^+^/H^+^ antiporter structure of yeast, the mechanism of transport can be only speculated about, based on the structural analysis of bacterial homologs [43,44]. The latter revealed dimeric organization, with the interacting dimerization domains flanked with the transport domains moving in an elevator-like fashion to assist the transport of ions. Homologous human Nhe1 protein (~13% sequence identity with Cnh1) also revealed homodimeric organization [45] pointing to the fold conservation, so it is highly probable that yeast sodium/proton antiporters also form such dimers; however, to reveal all the details about the exact structure of pH sensor, proton-shuffling residues, and sodium binding sites, the structure of yeast antiporter is essential. Blocking the elevator movement of a transport domain with a small molecule might be a viable route for drug development targeting these transporters.

### 2.3. Potassium Transporting Systems

Potassium is an essential alkali metal for all kingdoms of life. For example, together with sodium, it is involved in the buildup of the membrane potential. Furthermore, it contributes to the regulation of intracellular pH and osmolarity, and can serve as a cofactor for some enzymes [42,46]. As much higher potassium concentrations are found intracellularly than extracellularly and the internal K^+^ concentration is maintained at a nearly constant level, the conclusion can be made that cells should host efficient potassium transport systems localized in their plasma membranes. *C. albicans* is not an exception here and it encodes three potassium transport systems in its genome. Those are Trk uniporters, Hak potassium-proton symporters, and Acu ATPases [47].

**Figure 3:**
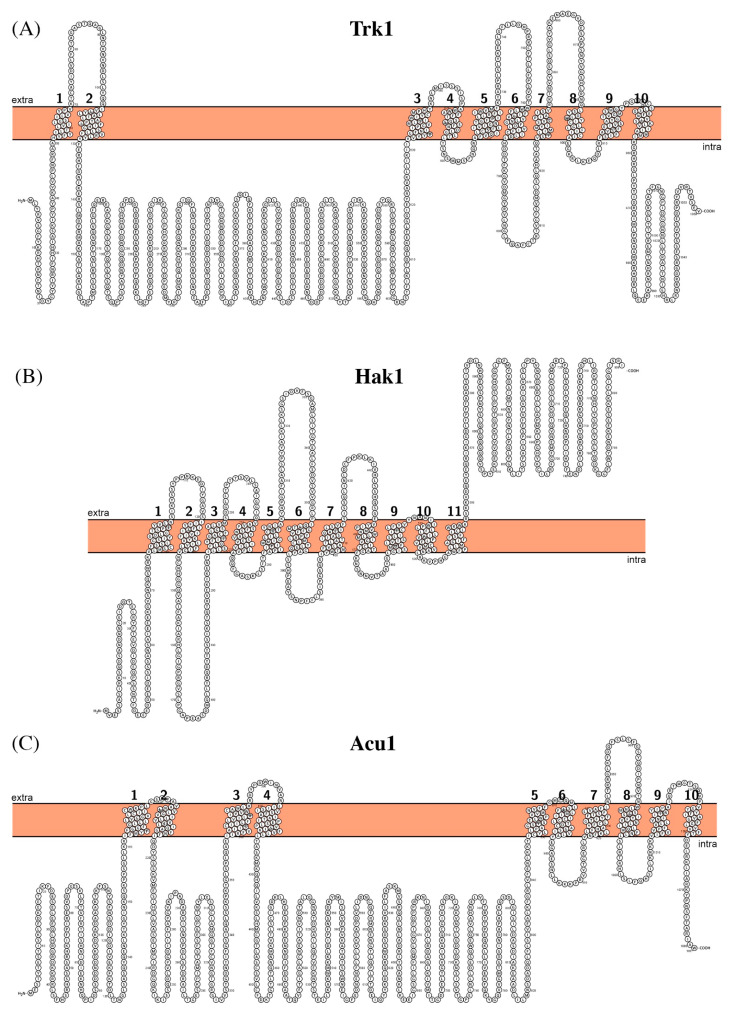
Schematic representation of the proposed secondary structure and topology of Trk1 (**A**), Hak1 (**B**), and Acu1 (**C**).

Trk1 of *C. albicans* belongs to the K^+^ Transporter (hence Trk) Family of proteins, present in both Gram-negative and Gram-positive bacteria, yeast, and plants. It is a large plasma membrane protein made of 1056 amino acid residues (Mw ~120 kDa) with ten predicted transmembrane segments (Figure 3A) and presumably with both N- and C-termini inside. Based on comparison of C-termini of KcsA from *Streptomyces lividans* and Trk1 from fungi, the proposal was made that some segments of Trk1 protein could be formed by duplicating segments from a common ancestor [48]. Based on the sequence analysis and homology modelling, the following conserved architecture of fungal Trk proteins has been proposed: single polypeptide chain of Ktr folds into a homodimer of four joint units each, where each unit (or MPM motif [49]) resembles a canonical potassium channel KscA [50] and consists of two transmembrane helices, M1 and M2, connected via the pore helix and the selectivity filter loop with an essentially conserved Gly residue [51].

Trk1 was shown to be an essential gene for *C. albicans*, and its product was proposed as the major potassium transport protein in this species, especially when the external potassium concentrations are low [52]. Furthermore, it was proposed as an effector of candidacidal activities of antimicrobial peptides such as histatin 5, enhancing its toxic effect to *C. albicans* cells [53]. Trk1 of *C. albicans* is functionally similar to Trk1 of *S. cerevisiae* (sequence identity ~32.7%), with the main function of K^+^ import, albeit with a lesser selectivity (potassium over sodium) than in canonical potassium channels. The second important function of this protein is the efflux of chloride ions, which might be necessary for chloride detoxification in high salt environments [54].

The proposed mode of transport is uniport, driven by the membrane potential [52].

Currently, there is no experimentally-derived structure of yeast Trk1 protein available, apart from the modelled structure of *S. cerevisiae* yeast Ktr1 [51]. Although it provides some link between the proposed structure and available experimental data, it still cannot explain all the observations fully, as for example the structure of the large cytosolic domain (~500 amino acid residues) is still unknown.

In addition to constitutively expressed Trk1, *C. albicans* also possesses inducible high-affinity K^+^ (HAK) transporter [55,56]. It is 808 amino acid residues long (Mw ~90 kDa) with the 11 predicted transmembrane segments (Figure 3B). In contrast to Trk1 proteins, HAK transporters are not ubiquitous and are mostly found in yeast species experiencing low potassium environment or K^+^ starvation [57,58,59]. Functional studies on homologs have shown that it is also able to transport Rb^+^ and that the transport is coupled to H^+^ [60]. It is also distantly related to bacterial K^+^ Uptake Permease (KUP) Family of proteins, which are present in both Gram-negative and Gram-positive bacteria [61,62]. Recently, a structure of KUP family transporter KimA from *Bacillus subtilis* has been reported, which revealed the mechanism of K^+^/H^+^ symport. KimA operates in the alternating fashion, where the outward-open conformation allows entry of K^+^ and H^+^ from the extracellular environment. Binding of substrates causes the movement of the gate tyrosine residue followed by the rearrangement of transmembrane helices 1 and 6, ultimately leading to the opening of the pore towards the cytoplasm and its closure at the extracellular side. The protonation of conserved glutamate residue leads to the opening of the intracellular gate followed by K^+^ release towards cytoplasm [63]. The sequence identity between KimA of *B.subtilis* and Hak1 of *C. albicans* is only ~11%, but intriguingly, the proton-binder glutamate residue is also conserved in *C. albicans*. However, only with the actual structure of Hak1 will it be possible to make the conclusion of whether the same mechanism is utilized in yeast.

*C. albicans* is one of the few yeast species that encodes an additional potassium transporter system—Acu1 ATPase. The gene encoding Acu1 in the canonical SC5314 strain contains a stop codon, which, if replaced, restores open reading frame (ORF) and the full-length protein of 1081 amino acid residues long can be produced [64]. It has 10 putative transmembrane segments with a long intracellular loop between segments 4 and 5, which contains an ATP-binding site (Figure 3C). Importantly, in two-thirds of analyzed *C. albicans* strains, ORF encoding Acu1 seems to be unbroken [64].

It has been shown that the full-length Acu1 of *C. albicans* has a high affinity for potassium ions and greatly improves the tolerance to lithium ions, but it does not transport sodium ions [64]. Furthermore, it can also modulate membrane potential or pH to some extent [65]. The exact functional role of this protein is still puzzling, but apparently in contrast to the house-keeping Trk1 protein, Acu1 together with Hak1 are expressed under stress, such as altered pH environments and in very low K^+^ concentrations.

As potassium uptake and its accumulation are essential for *C. albicans* cell growth, the aforementioned transporters may serve as novel targets for the development of new antifungal drugs.

### 2.4. Calcium Transport Systems

Ca^2+^ is an essential divalent ion, playing important functions in eukaryotic cells [66,67]. One of the major processes where calcium is recruited as a messenger molecule is calcium signaling [68]. It is used for cell-to-cell communication, as an allosteric regulator for numerous enzymes and to trigger specific cellular responses. To make such signaling efficient, the intracellular calcium concentration is tightly regulated and kept at low values of ~100 nM. This requires an orchestrated action of plasma membrane and organelle transport systems, such as calcium pumps and exchangers to extrude the surplus of calcium away from cytoplasm, but also calcium channels to get it into cytoplasm, when necessary. Here, we will describe only a few calcium transport systems located in the plasma membrane of *C. albicans* that have been characterized up to date.

*High affinity Ca^2+^ uptake system (HACS)* of *C. albicans* includes proteins Cch1, Mid1, and Ecm7. This is the main gateway for Ca^2+^ influx into cells [69,70,71,72,73,74]. Interestingly, Cch1 and Mid1 seem to form a complex, where Cch1 forms a channel and Mid1 is a regulatory subunit [75] in the absence of which the channel function is lost [76]. Cch1 and Mid1 are homologous to the catalytic and regulatory subunits of mammalian voltage-gated calcium channels, respectively [70,77]. Ecm7 belongs to the PMP-22/EMP/MP20/Claudin superfamily of transmembrane proteins [69,70,72,78], and its main function is the regulation of Cch1-Mid1 complex [72,79,80].

**Figure 4:**
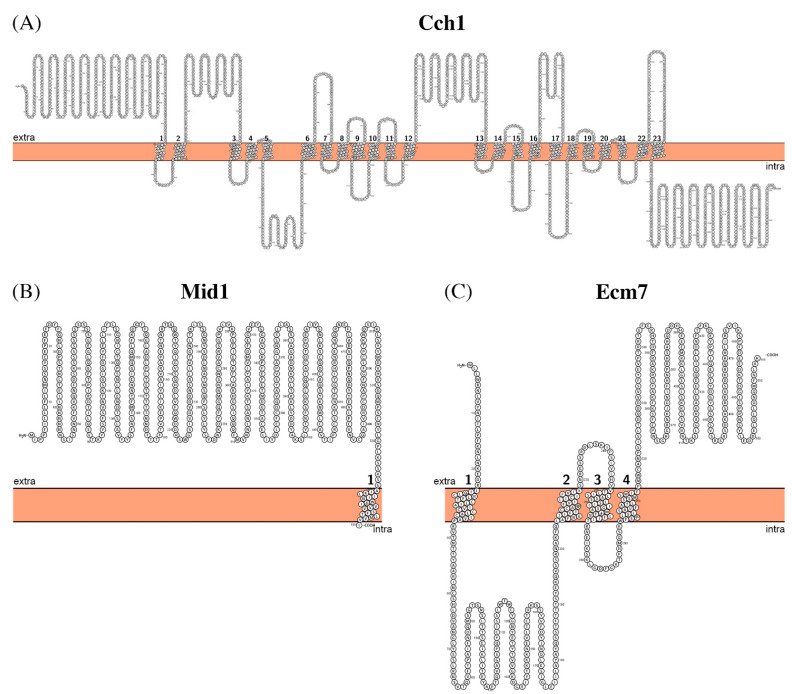
Schematic representation of the proposed secondary structure and topology of Cch1 (**A**), Mid1 (**B**), and Ecm7 (**C**).

Cch1 of *C. albicans* is a very large membrane protein of 2254 amino acid residues long (Mw ~260 kDa) with at least 23 predicted transmembrane helices (Figure 4A). Mid1 is 559 amino acid residues long (Mw ~63 kDa) with one predicted transmembrane segment (Figure 4B); hence, supposedly, it should interact with the extracellular part of Cch1. Ecm7 has 514 amino acid residues in its sequence (Mw ~58 kDa) with four transmembrane segments (Figure 4C). No structures are currently available for any of these proteins, so the exact modes of interactions among them remain unclear.

HACS has evolved as an efficient adaption and defense system in yeast and is triggered by oxidative stress [81], changes in pH [70], azole-class antifungal agents [77,82,83], and other xenobiotics.

The Ca^2+^ influx via HACS leads to the activation of calcineurin [84,85], which in turn activates a calcium antiporter and calcium pump [75,86].

It has been shown that the deletion of Cch1 or Mid1 in *C. albicans* affected hypha formation and maintenance, invasive growth, and sensitivity to oxidant agents, and significantly attenuated the virulence of *C. albicans* in vivo in a mouse model [75,87]. The main reason for that could be an inability of mutants to sustain the continuous activation of Ca^2+^/calmodulin signals, which results in the switch from hyphal to pseudohyphal morphology. Therefore, targeting these channels is a promising route for the development of new antifungals.

In addition to HACS, there is also the low-affinity calcium system (LACS), which mainly consists of mating factor-induced gene 1 (Fig1) channel and Rch1 regulator. Fig1 facilitates calcium influx and cell fusion during mating and was shown to be upregulated in response to mating pheromones in both *S. cerevisiae* and *C. albicans* [88,89,90]. Rch1 is a novel negative regulator of calcium uptake via Fig1 [91,92].

**Figure 5:**
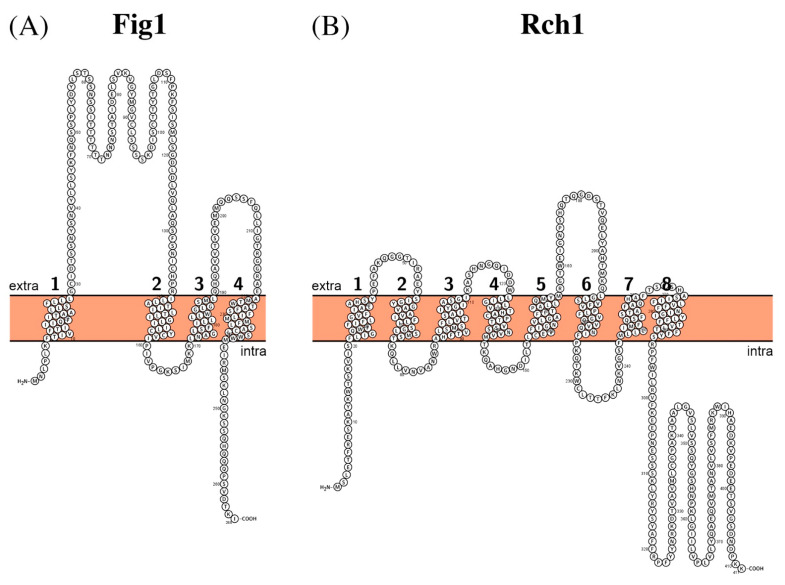
Schematic representation of the proposed secondary structure and topology of (**A**) and Rch1 (**B**).

Fig1 is a short membrane protein of 265 amino acid residues long (Mw ~26 kDa) and contains four predicted transmembrane segments (Figure 5A) with a loop between the first and second TM segments that is expected to be extracellular and contain several potentially glycosylated residues [93].

During the mating process of yeast cells, diploid *C. albicans* cells must become homozygous at the mating-type locus and switch from white yeast-shaped cells to mating-competent, bean-shaped opaque cells [94]. Mating-competent cells form shmoo mating projections and, in this process, pheromone-dependent chemotropism can occur, which stabilizes chemotropic gradients and facilitates the directed growth of mating projections toward each other, then followed by cells’ fusion [95]. To maintain the directionality of the growth, the influx of calcium ions is necessary [96], and this is where Fig1 is recruited for regulating calcium ion uptake at sites of polarized tip growth [90] to enhance membrane stability during morphological transitions. Deletion of Fig1 gene leads to significantly reduced ability of hyphal tips to reorient upon contact with ridges, but it does not affect Ca^2+^ uptake during vegetative growth [97,98].

Recently, the Regulator of Ca^2+^ homoeostasis 1 (Rch1) protein has been proposed as a regulator of LACS [91] without evidence for the direct interaction with HACS system [92], which, however, was challenged later by Xu et al. [99], who showed that HACS and Rch1 are epistatic.

Rch1 contains 411 amino acid residues (Mw ~46 kDa) and is homologous to human SLC10A7 (ssolute carrier family 10 member 7) sodium/bile acid transporter with 22.6% sequence identity. It has eight predicted transmembrane segments with both C- and N-termini inside the cell (Figure 5B). This protein is also known to be a cytosolic Ca^2+^ regulator. The temporary increase of Ca^2+^ level in cytosol activates Ca^2+^/calcineurin signaling pathway necessary for cells’ proper stress response [91,100].

Deletion of Rch1 leads to calcium hypersensitivity and tolerance to azoles and Li^+^ [91]. It seems that Rch1 functions as a negative regulator of cytosolic Ca^2+^ homeostasis by a rapid feedback inhibition of the Ca^2+^ influx.

Clearly, more functional and especially structural studies are necessary to decipher the intricate calcium homeostasis in *C. albicans*.

## 3. Heavy Metals

### 3.1. Iron Transport Systems

Iron is an essential metal required for numerous metabolic pathways. It has a limited bioavailability for most organisms and, in many cases, it is required for pathogenicity and virulence of microorganisms [101,102,103]. In the frame of pathogen–host interactions, there is a constant competition for iron, which led to sophisticated strategies in both hosts and pathogens for iron acquisition [104,105,106,107]. This is compounded with the poor solubility of ferric (Fe^3+^) ion near physiological pH and the fact that all iron is typically complexed and not present in the free form, as it can become very toxic as it catalyzes the production of reactive oxygen species [108].

In the particular case of *C. albicans*, iron has been shown to be an essential factor for its proliferation, survival, and virulence [109,110,111,112].

One of the most common host strategies is to reduce the availability of iron to microorganisms [113,114], and *C. albicans* has acquired an intricate system to scavenge iron [112,115] from the environment. It can be done via three major pathways: reductive iron uptake system, siderophore uptake system, and hemoglobin uptake system [116].

A reductive iron uptake system consists of a high-affinity iron permease Ftr1 [117], which is responsible for iron import [118,119] and is essential for systemic infection. It also has a homologue Ftr2 with the similar affinity to iron; however, it is not required for systemic infection and is regulated in the opposite way to Ftr1; namely, expression of Ftr1 is induced and that of Ftr2 is repressed during iron depletion, and vice versa in iron-rich medium [117]. This ensures inflow of iron at the variety of conditions to which *C. albicans* might be exposed.

**Figure 6:**
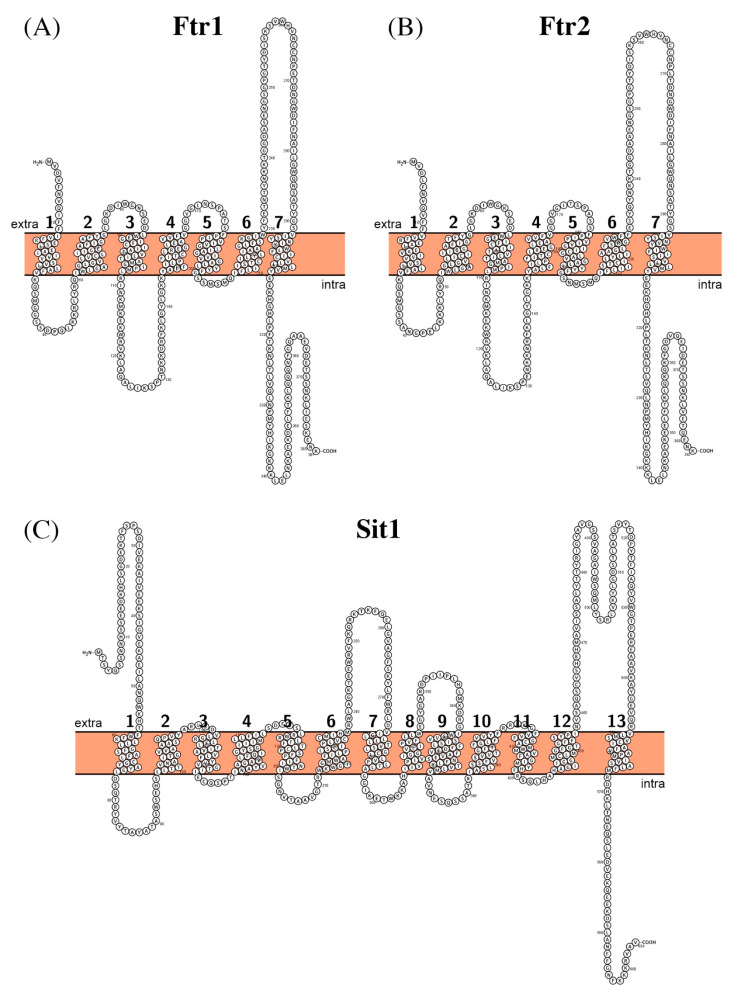
Schematic representation of the proposed secondary structure and topology of Ftr1 (**A**), Ftr2 (**B**), and Sit1 (**C**).

Ftr1 and Ftr2 are 381 and 382 amino acid residues long (Mw ~42.5 kDa), respectively, with the sequence identity of ~85%, hence it is not surprising that both proteins have similar affinities for iron. Both proteins show seven predicted transmembrane segments and are likely to have a very similar structure (Figure 6A,B).

However, Ftr1 and Ftr2 are not functional on their own and must associate with a ferroxidase to form a functional system [120,121,122,123]. The role of these ferroxidases is to oxidize the ferrous ion (Fe^2+^) to Fe^3+^, which then will be transported via Ftr1 or Ftr2 [101]. *C. albicans* encodes five different ferroxidases FET3, FET31, FET33, FET34, and FET99, which, in combination with plasma membrane Ftr1 and Ftr2 permeases as well as vacuolar Fth1 and Fth2 permeases, might form 20 different complexes [124]. It turned out that all but Fet33 can form complexes with both plasma membrane and vacuolar proteins with Fet33 specific for the latter [124]. This suggests that there are eight possible complexes in the plasma membrane, which is a possible corollary of the commensal nature of *C. albicans*, which requires adaptability to wide concentrations of iron in different host niches.

Such a complex of ferroxidase-permease for iron uptake is unique to fungi (and algae); hence, drugs targeting this system would have less off-targets in humans.

The other pathway for *C. albicans* is to sequester iron via siderophores, small molecules capable of chelating iron with very high affinities. Interestingly, *C. albicans* is not able to produce its own siderophores; however, it has acquired an ability to utilize those produced by other fungi and bacteria [112,125]. The iron-loaded siderophore uptake in *C. albicans* is driven by Sit1 (also termed Arn1) siderophore transporter.

It is 604 amino acid residues long (Mw ~67 kDa) and has 13 predicted transmembrane helices (Figure 6C). It belongs to the Siderophore-Iron Transporter family, which is a part of the major facilitator superfamily. Members of Sit family are found in fungi and most probably proton-coupled symporters [126,127,128,129,130].

Sit1 of *C. albicans* has been shown to be involved in the uptake of hydroxamate-type siderophores, such as ferrichrome [131], but also ferricrocin, ferrichrysin, and ferrirubin and to some extent of triacetylfusarinine and coprogen [132].

In addition, *C. albicans* has several genes encoding ferric reductases, which are necessary to reduce Fe^3+^ bound to siderophores [133].

Finally, *C. albicans* can bind to and lyse erythrocytes and sequester iron from heme [134,135,136,137]. This is done via the secretion of proteinases, which are able to degrade heme-containing proteins including hemoglobin [138]; transferrin seems to be yet another source of iron, permitting growth of *C. albicans* in the blood stream [139].

### 3.2. Zinc Transporting Systems

Zinc is another essential microelement, widely recruited in the cell as a cofactor for numerous proteins [140]. However, it is also quite toxic, hence cells have evolved to tightly regulate zinc homeostasis and its transport inside and outside the cell [141,142,143]. The consequence of this is a very low concentration of free Zn^2+^ as it is normally complexed within the cell. Therefore, in the host–pathogen war, the host is trying either to limit the access to valuable metal cations or to increase the metal concentration to toxic levels [141].

To withstand such extremities, *C. albicans* is equipped with high-affinity zinc importers and exporters. Furthermore, Zn^2+^ is crucial for zinc-binding proteins involved in fungal virulence [144].

For efficient Zn^2+^ import, it has two homologs of Zip (Zrt-, Irt-like Protein) family, namely Zrt1 and Zrt2, located in the plasma membrane. Zip family transporters are found in all kingdoms of life, and some members are iron rather than zinc transporters [145,146,147].

**Figure 7:**
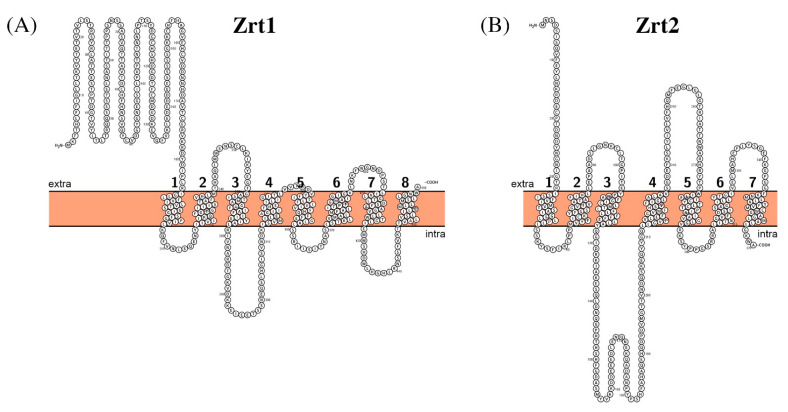
Schematic representation of the proposed secondary structure and topology of Zrt1 (**A**) and Zrt2 (**B**).

Zrt1 and Zrt2 are 468 (Mw ~51 kDa) and 370 (Mw ~41 kDa) amino acid residues long, respectively, and each is predicted to have at least seven transmembrane segments (Figure 7). These proteins share only ~20% sequence identity. It seems that the presence of two homologs is necessary to maintain viability (and virulence) at the broad pH range, stressing again the versatility of *C. albicans* in terms of adaptation; Zrt2 is the main importer at acidic pH and Zrt1 is functional at pH 7 and above [148]. These proteins are proposed to operate as secondary active transporters, although the mechanism of transport is not clear in the absence of structural and functional information.

The uptake of zinc is assisted by an extracellular zincophore protein, pH-regulated antigen 1 (Pra1), which sequesters zinc ions with very high affinity [149,150]. It is heavily glycosylated and may interact with fibrinogen via O-linked sugars. Together with surface mannoprotein 65 and Hyphally-regulated protein Hyp1, they contribute to the carbohydrate component of the biofilm matrix. Pra1 was also shown to mediate leukocyte adhesion and migration [151].

Taking into account the critical role of zinc in the virulence of such fungal pathogens as *C. albicans, Aspergillus fumigatus, Histoplasma capsulatum, Cryptococcus neoformans,* and *Cryptococcus gattii* [152,153,154,155], inhibition of zinc uptake systems seems to be a promising route for drug development.

### 3.3. Copper Transporting Systems

Similarly to zinc and iron, copper is an essential microelement, recruited by numerous enzymes that utilize the redox properties of copper [156]. It is commonly found in enzymes involved in essential biological processes such as respiration, iron acquisition, and protection against oxidative stress. The bioavailability of copper is also low, as it mainly exists in the form of insoluble complexes. To further compound these issues, it is not only toxic in high concentrations, but it also can generate destructive hydroxyl radicals from hydrogen peroxide [157]. This strongly suggests that the transport and homeostasis of copper should be tightly regulated.

Furthermore, copper has been shown to be critical for the virulence of many pathogens, including *C. albicans* [158,159,160]. It is also one of the key elements in the framework of host–pathogen interactions, where the host either sequesters it away, limiting the availability of copper to pathogens, or increases its concentration to embody copper toxicity [141,161].

The main copper uptake system of *C. albicans* is a plasma membrane protein Ctr1 [162], whose expression is upregulated in the copper-limiting conditions through metal binding activator 1 (Mac1) [163]. Furthermore, its expression seems to be pH-dependent and is induced during growth in alkaline pH via the pH-responsive transcription factor, Rim101 [164].

**Figure 8:**
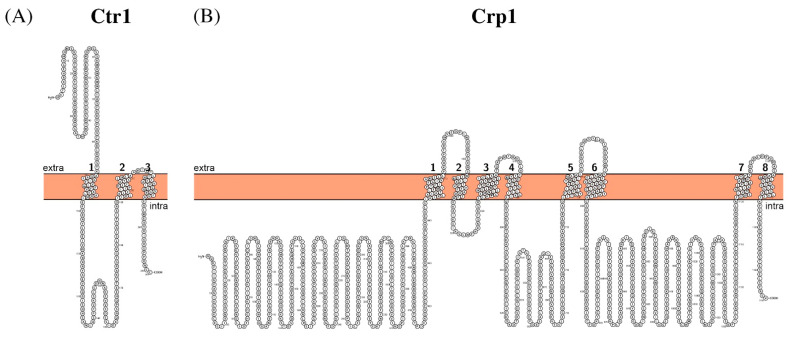
Schematic representation of the proposed secondary structure and topology of Ctr1 (**A**) and Crp1 (**B**).

It is a relatively small protein of only 251 amino acid residues long (Mw ~28 kDa) with only three predicted transmembrane segments (Figure 8A). The human homologue was shown to form functional trimers with the pore in the middle to conduct copper and silver ions as well as platinum-based drugs [165,166,167]. The mechanism of transport seems to be a passive diffusion [166]; however, copper ions are almost immediately bound to acceptor proteins such as intracellular chaperones and low molecular weight chelators [168,169].

Recently the manganese transporter Smf12 was proposed as a putative copper transporter as its expression was enhanced during copper starvation and it is also under regulation of Mac1 [170]. The same role of copper transport for Smf12 was suggested in *S. cerevisiae* [171].

Interestingly, copper deficiency also impacts iron homeostasis, as iron uptake depends on the multicopper ferroxidases (Fre proteins, see above). However, an excess of copper is also detrimental for iron homeostasis, as it triggers copper detoxification, hence the iron uptake machinery is ultimately affected as well [172].

To survive an elevated toxic concentration of copper, *C. albicans* chelates intracellular copper by a specific metallothionein Cup1 and expels it via a plasma-membrane P-type ATPase pump Crp1. Both proteins play a role in the protection of *C. albicans* against oxidative stress [173].

Crp1 is a large multi-domain protein of 1197 amino acid residues (Mw ~132 kDa) with eight transmembrane segments in the membrane section (Figure 8B).

The only resolved copper-specific P-type ATPase CopA is from bacterium *Legionella pneumophila* [174], which has eight transmembrane helices, ~500 amino acid residues shorter, and shares ~19% sequence identity with Crp1 of *C. albicans*. Interestingly, the residues shown to coordinate copper ions in CopA are invariantly conserved in Crt1, hinting towards the possibility of the evolutionary well-conserved copper detoxification mechanism. Still, the exact details of this mechanism in *C. albicans* are obscure in the absence of Crt1 structure.

## 4. Concluding Remarks

*C. albicans* is an insidious commensal microorganism that is capable of adapting to very different environmental niches within the body. It is the prevalent opportunistic fungal invasive pathogen of severe and quite often fatal infections. Currently, there is a surge in antibiotic-resistant strains of *C. albicans*, and it is the right moment to start developing new drugs targeting this pathogen. Ion homeostasis is crucial for any living cell, and its disruption is usually fatal; thus, blocking ion fluxes seems to be a promising route for such development. As we summarized in this review, there are several promising targets (some of which are rather unique, such as Ftr1 (Ftr2)-ferroxidases complexes); however, most of them are poorly characterized structurally. Future structural work on the aforementioned proteins will certainly boost the development of novel medications to control the spread of this important pathogen.

## Figures and Tables

**Table 1:** Unique identifiers for proteins discussed in this manuscript.

Protein	Uniprot Accession Number	Candida Genome Database Accession Number	Transporter Classification Database Identifier
Mep1	A0A1D8PJF2	C3_02310W	1.A.11.3
Mep2	Q59UP8	C4_00430W	1.A.11.3
Cnh1	Q9P937	C4_00040W	2.A.36.4.4
Trk1	A0A1D8PTL7	CR_07960C	2.A.38.2
Hak1	A0A1D8PDU7	C1_06610C	2.A.72.2
Acu1	Q5A9B2	CR_01640C	3.A.3
Cch1	Q5A936	C1_01100W	1.A.1.11
Mid1	A0A1D8PNU4	C5_03990W	8.A.41.1
Ecm7	Q59US7	C4_00180W	1.H.1.4
Fig1	Q59WR6	C6_01310W	1.A.81.2.1
Rch1	Q59UQ7	C4_00360C	2.A.28.3.7
Ftr1	A0A1D8PFV0	C1_14130W	2.A.108.1.2
Ftr2	A0A1D8PFV2	C1_14220C	2.A.108.1.3
Sit1	Q5A2T6	C2_08050C	2.A.1.16
Zrt1	A0A1D8PMR6	C4_06970C	2.A.5.1
Zrt2	A0A1D8PGN5	C2_02590W	2.A.5.1
Ctr1	Q59NP1	C6_00790C	1.A.56.2.2
Crp1	A0A1D8PEI4	C1_09250W	3.A.3.5.9

## Methodology

Protein sequences were extracted from the curated “Candida Genome Database” http://www.candidagenome.org/ (accessed on 1 February 2021) and corresponding structures, if available, from Protein data bank https://www.rcsb.org (accessed on 1 February 2021). The transmembrane topology for all proteins discussed in the manuscript was predicted via Phobius (https://www.ebi.ac.uk/Tools/pfa/phobius/) (accessed on 1 February 2021) and visualized with Protter 1.0 (https://github.com/ulo/Protter) (accessed on 1 February 2021). No filtering was applied to the literature sources.
